# Supplementary figures and images for: Factors associated with dyslipidemia among healthcare workers in a COVID-19-designated hospital in Hangzhou, Zhejiang, China: A retrospective cohort study from 2019 to 2022
Source: PLoS One. 2025 Jun 30;20(6):e0323934. doi: 10.1371/journal.pone.0323934 (PMC12208422; doi:10.1371/journal.pone.0323934)

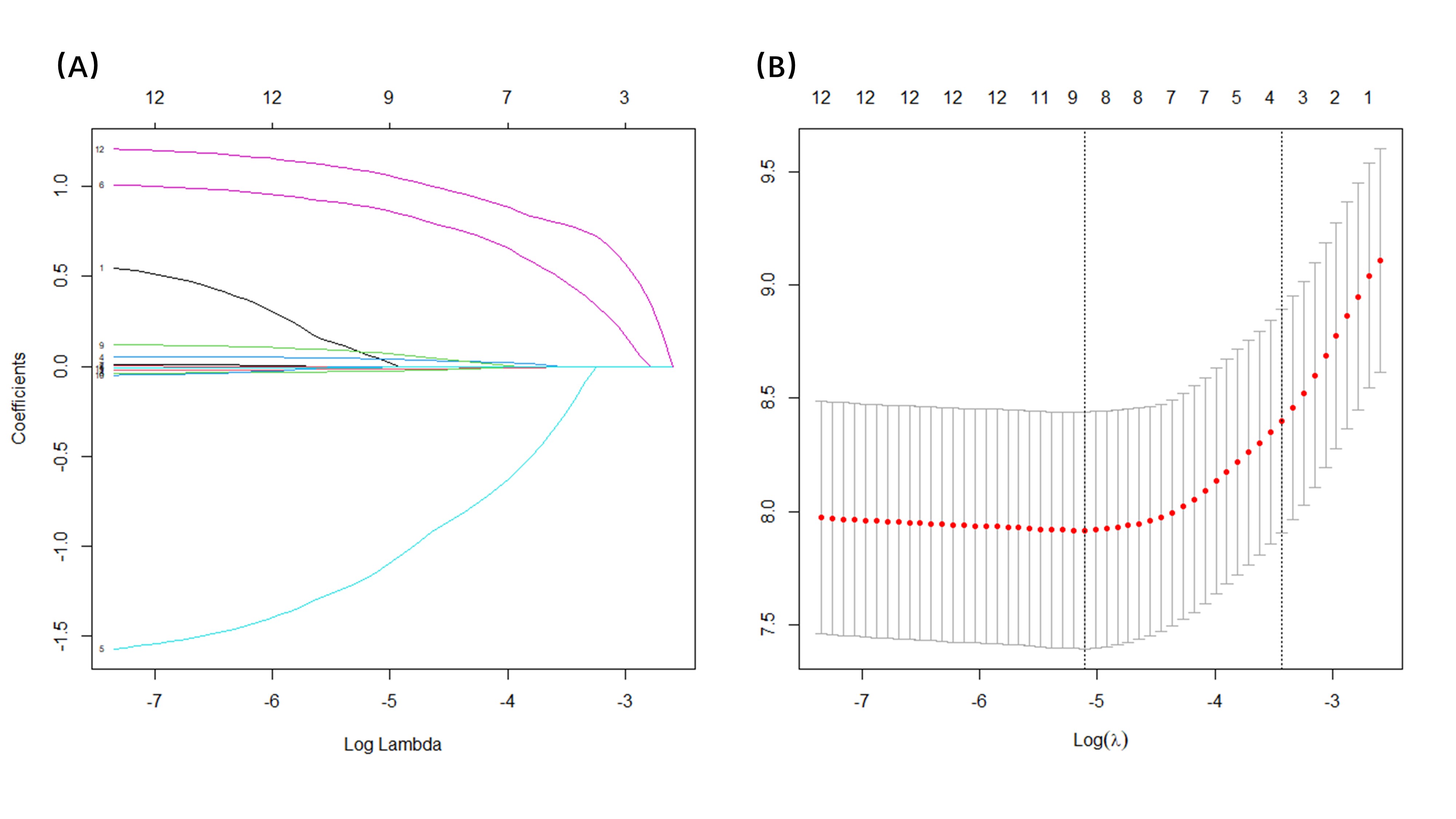

Supplement: S1 Fig — (A) Plot of partial likelihood deviance (Each color curve represents the LASSO coefficient profile of a variable against the Log (λ) sequence.); (B) plot of LASSO coefficient profiles (The values above the figure represent the numbers of variables included in the model, with the corresponding λ shown on the x-axis; λ: lambda). (JPG) [file pone.0323934.s001.jpg]
