# Supplementary material for: Factors associated with dyslipidemia among healthcare workers in a COVID-19-designated hospital in Hangzhou, Zhejiang, China: A retrospective cohort study from 2019 to 2022
Source: PLoS One. 2025 Jun 30;20(6):e0323934. doi: 10.1371/journal.pone.0323934 (PMC12208422; doi:10.1371/journal.pone.0323934)
Supplement: Attachment — Submitted filename: Respond to Reviewer 2.docx [file pone.0323934.s004.docx]

Reviewer #2: Zuo et al. investigated the prevalence and determinants of dyslipidemia among healthcare workers during the COVID-19 pandemic in "Factors associated with dyslipidemia among healthcare workers in a COVID-19-designated hospital in Hangzhou, Zhejiang, China: a retrospective cohort study from 2019 to 2022". The study found that healthcare workers with significant frontline working time and longer previous working time were less likely to develop dyslipidemia. I believe the manuscript could be further improved by incorporating the following edits:

1. Page 9, Lines 62-67: Please provide a reference to support these statements.

**Answer:** We would like to thank you for your critical and constructive comments. The statements were from Ref 9-10, and we added them in the manuscript.

2. Page 10, Lines 80-82: Avoid using vague and imprecise terms like “normal people”. Instead, it would be better to compare front line workers to specified reference groups such as the general population or other relevant cohort to provide a clearer context for comparison.

**Answer:** We would like to thank you for your critical and constructive comments. We changed the “normal people” into “general people” and checked the manuscript to avoid these vague and imprecise terms.

3. Page 13, Line 134: It is unclear what the authors mean by the term “authorized strength” in the context of the study. Are they referring to staffing levels, qualifications or something else. Please define this term when first mentioned in the text to avoid any ambiguity.

**Answer:** We would like to thank you for your critical and constructive comments. And we explain the authorized strength in the manuscript to help readers better understand the context of medical staff. Here is the explanation: “Medical staff are divided into those with authorized strength or not, and those with authorized strength tend to have high social status, decent pay, and good social welfare.”

4. Page 13, Lines 137-140: The use of the phrase “month of examination of 2022’ when evaluating frontline working time is very vague. To improve transparency and avoid any confusion, the authors should clearly explain how they calculated the time period for medical staff with normal lipid levels.

**Answer:** We would like to thank you for your critical and constructive comments. We explain the definition with an example to help readers better understand. Here is the detail, “For example, a female healthcare worker had normal blood lipids from 2020 to 2022, and her physical examination in 2022 was done in August, so her frontline work time was accumulated from January 2020 to August 2022.”

5. Page 15, Line 180: Replace the period in ‘(30.41)’ with comma when reporting median value.

**Answer:** We would like to thank you for your attentiveness and seriousness. This is a typing error, and we have corrected it in the manuscript.

6. Pages 22-23, Lines 288-290: When citing the study on work-family conflict, please provide specific results, such as percentages or statistical findings, this will help the readers better understand the comparison and improve the validity of the discussion.

**Answer:** We would like to thank you for your critical and constructive comments. We rewrite this paragraph with “Research (23) found that healthcare workers with 1-5, 6-10, and ≥11 years of service had 1.78 (1.61-1.96), 2.31 (2.10-2.53), and 2.43 (2.18-2.69) folds to experience work-family conflict compared with <1 year of service, which in turn made them better at handling these conflicts under China’s prevention and control strategy.

7. Page 23, Line 303: Although the writers have briefly mentioned the concept of "edge elevation" in the discussion, they have not provided enough contextual information on its significance. Adding more details about the concept, including its importance and relevance to understanding the results, would improve the clarity of the discussion.

**Answer:** We would like to thank you for your critical and constructive comments. We add more details about the “edge elevation” in the following paragraph. “Edge elevation of blood lipids but still within the normal range, so there is no need to take lipid-lowering drugs temporarily. Healthcare workers can lower blood lipids by changing their lifestyle habits, such as paying attention to a low-salt and low-fat diet, engaging in regular exercises such as running and skipping rope, and ensuring sufficient sleep time and good sleep quality. These measures can help healthcare workers control their blood lipid levels. If not taken seriously, blood lipids can escalate from “edge elevation” to dyslipidemia, and even hyperlipidemia, which significantly increases the risk of cardiovascular and cerebrovascular diseases.”
